# Three-dimensional iodine mapping quantified by dual-energy CT for predicting programmed death-ligand 1 expression in invasive pulmonary adenocarcinoma

**DOI:** 10.1038/s41598-024-69470-9

**Published:** 2024-08-07

**Authors:** Kazuki Yamagata, Masahiro Yanagawa, Akinori Hata, Ryo Ogawa, Noriko Kikuchi, Shuhei Doi, Keisuke Ninomiya, Yukiko Tokuda, Noriyuki Tomiyama

**Affiliations:** 1 Department of Diagnostic and Interventional Radiology, Graduate School of Medicine, Osaka University, 2-2 Yamadaoka, Suita, Osaka 5650871 Japan; 2 Future Diagnostic Radiology, Graduate School of Medicine, Osaka University, 2-2 Yamadaoka, Suita, Osaka 5650871 Japan

**Keywords:** Dual-energy CT, Extracellular volume, Non-small cell lung cancer, Immune checkpoint inhibitor, Programmed cell death 1- ligand 1, Non-small-cell lung cancer, Lung cancer

## Abstract

We examined the association between texture features using three-dimensional (3D) io-dine density histogram on delayed phase of dual-energy CT (DECT) and expression of programmed death-ligand 1 (PD-L1) using immunostaining methods in non-small cell lung cancer. Consecutive 37 patients were scanned by DECT. Unenhanced and enhanced (3 min delay) images were obtained. 3D texture analysis was performed for each nodule to obtain 7 features (max, min, median, mean, standard deviation, skewness, and kurtosis) from iodine density mapping and extracellular volume (ECV). A pathologist evaluated a tumor proportion score (TPS, %) using PD-L1 immunostaining: PD-L1 high (TPS ≥ 50%) and low or negative expression (TPS < 50%). Associations between PD-L1 expression and each 8 parameter were evaluated using logistic regression analysis. The multivariate logistic regression analysis revealed that skewness and ECV were independent indicators associated with high PD-L1 expression (skewness: odds ratio [OR]  7.1 [95% CI 1.1, 45.6], *p* = 0.039; ECV: OR 6.6 [95% CI 1.1, 38.4], *p* = 0.037). In the receiver-operating characteristic analysis, the area under the curve of the combination of skewness and ECV was 0.83 (95% CI 0.67, 0.93) with sensitivity of 64% and specificity of 96%. Skewness from 3D iodine density histogram and ECV on dual energy CT were significant factors for predicting PD-L1 expression.

## Introduction

Lung cancer accounts for about 18% of all cancer-related mortalities worldwide, and adenocarcinoma is the most common type of pathology^1^. Lung adenocarcinoma can be divided into four categories based on the pathological invasion: preinvasive lesions such as adenocarcinoma in situ (AIS), minimally invasive adenocarcinoma (MIA), invasive adenocarcinoma (IVA), and variants^2^. Systemic therapy using chemotherapy with or without radiation therapy is the standard frontline treatment for advanced IVA (stage IIIB-IV), but the prognosis is poor. Recently, an immune checkpoint inhibitor (ICI) has been offered to patients with programmed cell death 1- ligand 1 (PD-L1) expression. ICIs targeting the programmed cell death 1 (PD-1) pathway have shown impressive clinical performance in several cancers, including non-small cell lung cancer (NSCLC)^3^. Therefore, it is important to prove or predict PD-L1 and PD-1 expression. In recent clinical practice, tissue biopsy is essential to evaluate gene expression within tumors. However, the tissue within tumors is heterogeneous. Tissue biopsy has problems with accuracy and reliability because only a portion of the tissue can be sampled. In addition, biopsy is invasive for patients and carries a risk of complications. On the other hand, CT is a non-invasive and widely used method. In addition, CT images cover the entire tumor and characterize it.

Previous studies using dual-energy CT have reported that quantitative values such as iodine concentration, and slopes of the spectral attenuation curves were helpful for predicting the gene expression level^4,5^. In addition, texture features can be calculated from a given image region to characterize the region texture^6^ and they are often used for image analysis especially for medical images^7^. Recently, 3D iodine density histogram texture analysis on dual-energy CT is available, and it may provide objective and reproducible data compared with two-dimensional (2D) one.

The extracellular volume (ECV) can also be obtained from the equilibrium phase of dual-energy CT. ECV represents the intravascular space and extravascular extracellular volume fractions. Many studies have shown that ECV is a useful quantitative imaging biomarker for fibrosis of organs such as the liver and myocardia^8,9^. And lung adenocarcinoma cells are well known to interact with various cells of the surrounding microenvironment, such as fibroblasts. Activated fibroblasts within lung cancer, called cancer-associated fibroblasts (CAFs), have been reported to be associated with PD-L1 expression^10^.

We hypothesized that dual-energy CT can predict the expression level of PD-L1 and texture features using 3D iodine density histogram texture analysis and ECV on dual-energy CT may correlate to the expression level of PD-L1. The purpose of this study was to examine the association between quantitative data measured by dual-energy CT and expression of PD-L1 using immunostaining methods.

## Results

### Patient data

Table 1 summarizes the patient and tumor characteristics. The final study population consisted of 37 patients and 37 nodules. 22 were male and 15 were female, and the mean age was 71 years (range, 38–85 years). Of the 37 nodules, 15 were part-solid nodules and 22 were solid nodules. The mean total size of nodules was 25.7 mm (range, 10.0–60.0 mm) and the mean volume of nodules was 5.5 cm3 (range, 0.12–99.6 cm3). 11 had high PD-L1 expression (TPS ≥ 50%) and 26 had low or negative PD-L1 expression (TPS < 50%).

**Table 1:** Tumor characteristics and patient demographics.

Age (years)	71 (38–85)
Sex	
Male	22 (59%)
Female	15 (40%)
Histology	
Papillary predominant	19 (51%)
Acinar predominant	3 (8%)
Lepidic predominant	6 (16%)
Solid predominant	9 (24%)
Pathological T factor	
pT1a	4 (11%)
pT1b	12 (32%)
pT1c	13 (35%)
pT2a	6 (16%)
pT2b	0 (0%)
pT3	2 (5%)
Nodule consistency	
Part-solid nodule	15 (40%)
Solid nodule	22 (59%)
Total size of all nodules (mm) (n = 37)	25.7 (10.0–60.0)
Total size of part-solid nodule (mm) (n = 15)	28.1 (15.0–42.0)
Solid portion size of part-solid nodule (mm) (n = 15)	15.7 (5.0–35.0)
Total size of solid nodule (mm) (n = 22)	24.1 (10.0–60.0)
Volume of all nodules (mm3) (n = 37)	5457.9 (122.5–99,590.3)
Volume of part-solid nodule (mm3) (n = 15)	1510.9 (122.5–4105.6)
Volume of solid nodule (mm3) (n = 22)	8149.0 (398.1–99,590.3)
PD-L1 expression	
Positive (TPS ≥ 50%)	11 (30%)
Low or negative (TPS < 50%)	26 (70%)

Age, tumor size, and nodule volume are shown as median (interquartile range).

### Cutoff value for predicting the expression of PD-L1

Table 2 shows the cutoff values obtained by ROC analysis for each of the seven radiomics features and ECV. In calculating ECV, iodine density in the aorta was 42.2 ± 3.6 (100 ug/cc) and hematocrit was 39.6 ± 4.6%. Mean, skewness, and kurtosis have significant difference between score = 0 and score = 1.

**Table 2:** Quantitative values from 3D iodine density histogram and ECV and cutoff value of the 8 parameters.

Parameters	Mean ± SD	Cutoff value
Max (100ug/cc)	72.9 ± 33.7	≤ 71
Score = 0 (n = 18)	90.9 ± 39.9	
Score = 1 (n = 19)	55.8 ± 11.8	
Min (100ug/cc)	− 114.3 ± 18.7	≤ − 104
Score = 0 (n = 9)	− 91.9 ± 11.8	
Score = 1 (n = 28)	− 121.5 ± 14.2	
Median (100ug/cc)	26.3 ± 4.7	> 28
Score = 0 (n = 27)	24.0 ± 3.1	
Score = 1 (n = 10)	32.5 ± 2.1	
Mean (100ug/cc)	12.3 ± 5.3	> 13.9
Score = 0 (n = 23)	9.2 ± 3.9	
Score = 1 (n = 14)	17.5 ± 2.5	
SD (100ug/cc)	37.5 ± 9.3	≤ 36
Score = 0 (n = 22)	43.5 ± 6.5	
Score = 1 (n = 15)	28.6 ± 4.4	
Skewness	− 2.2 ± 0.8	≤ − 2.3
Score = 0 (n = 20)	− 1.6 ± 0.4	
Score = 1 (n = 17)	− 2.9 ± 0.5	
Kurtosis	7.5 ± 4.3	> 6.2
Score = 0 (n = 19)	4.2 ± 1.2	
Score = 1 (n = 18)	10.8 ± 3.6	
ECV (%)	17.5 ± 7.2	> 21.2
Score = 0 (n = 26)	14.2 ± 5.8	
Score = 1 (n = 11)	25.2 ± 3.1	

Optimal thresholds were determined for each variable separately using the Youden index to predict the high PD-L1 expression (TPS ≥ 50%).

The group that met the cutoff value condition was graded as score = 1 and the other group was graded as score = 0.

*ECV* extracellular volume, *TPS* tumor proportion score.

### Predicting performance for PD-L1 expression using quantitative value

**Figure 1:**
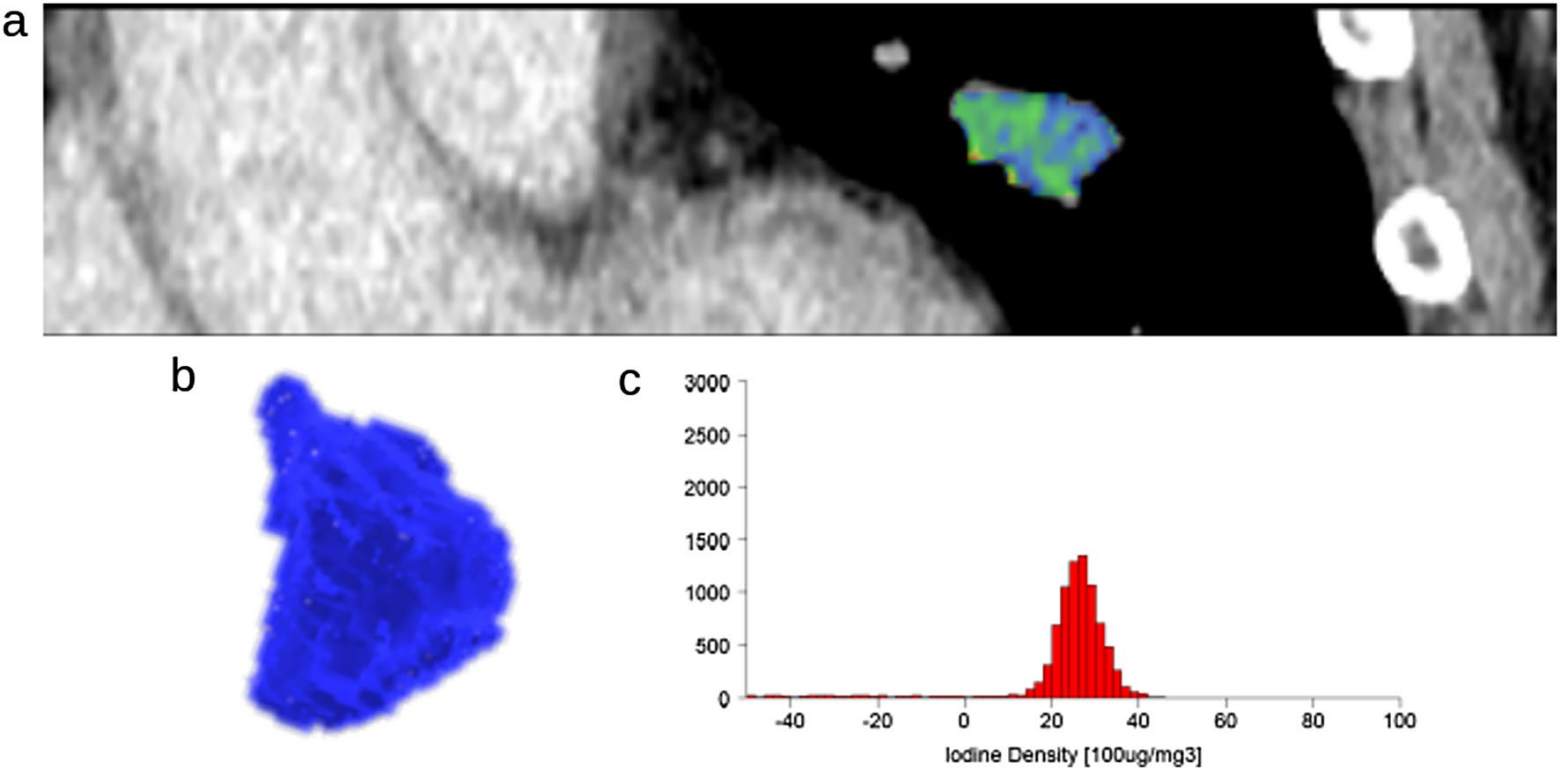
A representative case with PD-L1 negative invasive adenocarcinoma (TPS 0.5%). A coronal CT image with nodule segmentation (**a**), 3D volume of interest on tumor (**b**), and 3D iodine density histogram (**c**). 3D-iodine density mapping texture features and ECV were as follows: Max, 46 mg/cc; Min, − 153 mg/cc; Median, 26 mg/cc; Average, 13.88 mg/cc; Standard deviation, 38.34 mg/cc; Skewness, − 1.53; Kurtosis, 10.87; ECV, 17.57%. *PD-L1* programmed cell death 1- ligand 1, *TPS* tumor proportion score; ECV, extracellular volume.

**Figure 2:**
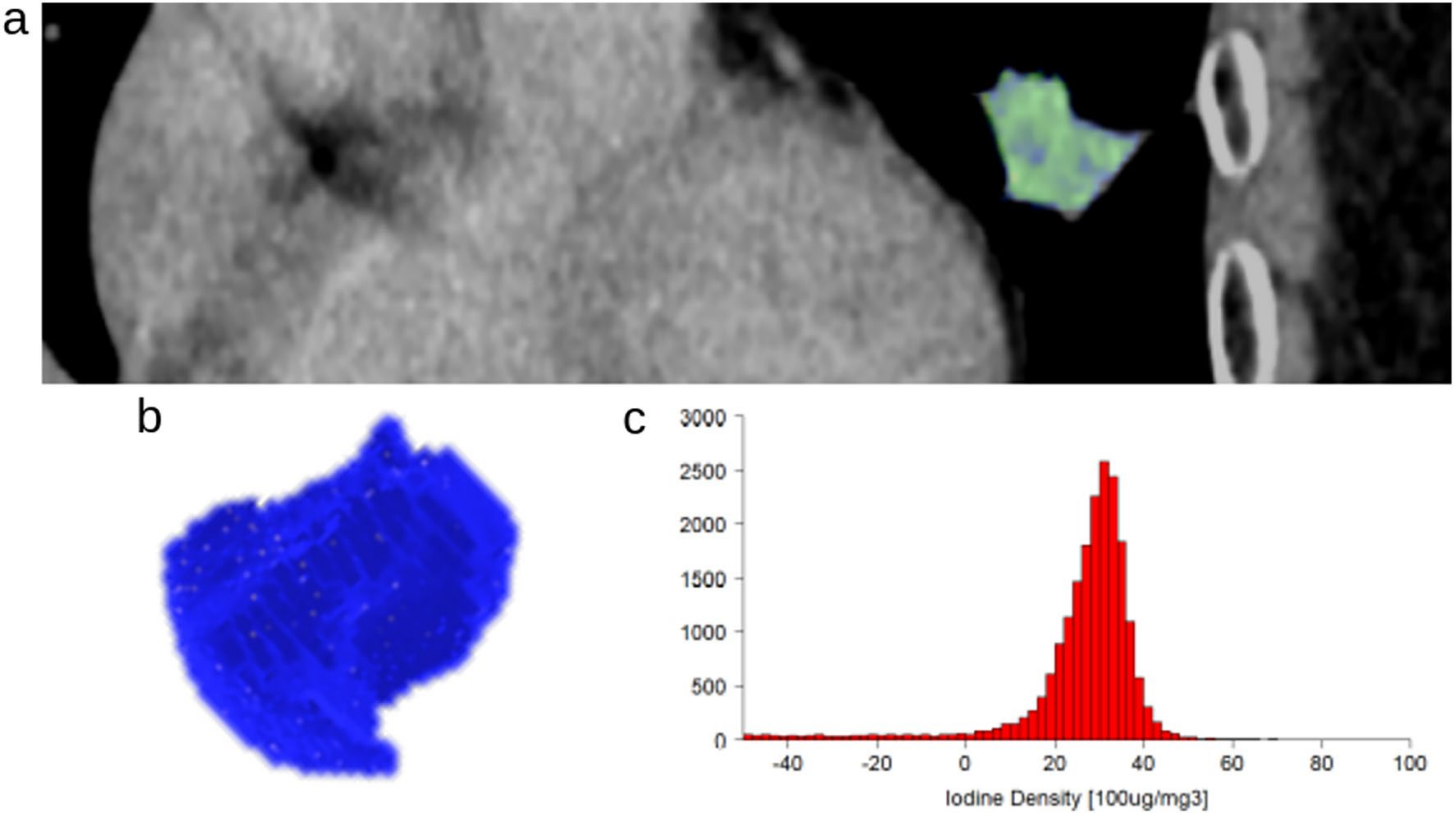
A representative case with PD-L1 positive invasive adenocarcinoma (TPS 80%). A coronal CT image with nodule segmentation (**a**), 3D volume of interest on tumor (**b**), and 3D iodine density histogram (**c**). 3D-iodine density mapping texture features and ECV were as follows: Max, 70 mg/cc; Min, − 108 mg/cc; Median, 29 mg/cc; Average, 20.5 mg/cc; Standard deviation, 28.82 mg/cc; Skewness, − 2.90 (cutoff value ≤ − 2.28); Kurtosis, 11.01; ECV, 27.60% (cutoff value > 21.23%). *PD-L1* programmed cell death 1- ligand 1, *TPS* tumor proportion score; ECV, extracellular volume.

Table 3 shows the result of logistic regression analysis. Univariate logistic regression analysis revealed that the mean, skewness, kurtosis, and ECV were significant indicators [odds ratio, 4.8, 10.1, 8.5, and 9.6; 95% confidence interval (CI) 1.1–21.3, 1.8–57.9, 1.5–48.0, and 1.9–48.9; *p* = 0.04, 0.009, 0.016, and 0.006, respectively]. Multivariate (bivariate) logistic regression analysis revealed that skewness and ECV were independent indicators associated with high PD-L1 expression [odds ratio, 7.1 and 6.6; 95% CI 1.1–45.6 and 1.1–38.4; *p* = 0.039 and 0.037, respectively]. With regard to the performance for predicting PD-L1 expression, AUC of the skewness and ECV for predicting PD-L1 expression were 0.76 and 0.74 (95% CI 0.58–0.88 and 0.57–0.87): sensitivity, 82% (9 of 11) and 64% (7 of 11) and specificity, 69% (18 of 26) and 85% (22 of 26), respectively. The AUC of the combination of skewness and ECV was 0.83 (95% CI 0.67–0.93): sensitivity, 64% (7 of 11) and specificity, 96% (25 of 26) (Table 4). Representative patients are shown in Figs. 1 and 2.

**Table 3:** Relationship of quantitative values with PD-L1 expression.

Quantitative values	Univariate analysis		Multivariate analysis with stepwise method	
	OR (95% CI)	*P* value	OR (95% CI)	*P* value
Max (n = 37)				
Score = 0 (n = 18)	3.6 (0.78, 16.9)	0.1		
Score = 1 (n = 19)				
Min (n = 37)				
Score = 0 (n = 9)	4.4 (0.48, 40.8)	0.18		
Score = 1 (n = 28)				
Median (n = 37)				
Score = 0 (n = 27)	3.5 (0.75, 16.3)	0.11		
Score = 1 (n = 10)				
Mean (n = 37)				
Score = 0 (n = 23)	4.8 (1.1, 21.3)	0.04*		
Score = 1 (n = 14)				
SD (n = 37)				
Score = 0 (n = 22)	3.9 (0.89, 17.4)	0.07		
Score = 1 (n = 15)				
Skewness (n = 37)				
Score = 0 (n = 20)	10.1 (1.8, 57.9)	0.009*	7.1 (1.1, 45.6)	0.039*
Score = 1 (n = 17)				
Kurtosis (n = 37)				
Score = 0 (n = 19)	8.5 (1.5, 48.0)	0.016*		
Score = 1 (n = 18)				
ECV (n = 37)				
Score = 0 (n = 26)	9.6 (1.9, 48.9)	0.006*	6.6 (1.1, 38.4)	0.037*
Score = 1 (n = 11)				

*OR* odds ratio, 95% CI 95% confidence interval.

*P values of < 0.05 were considered significant.

**Table 4:** Performance for predicting PD-L1 expression.

	Sensitivity	Specificity	Area under the curve (AUC)
Skewness	82% (9 of 11)	69% (18 of 26)	0.76 (95% CI 0.58–0.88)
ECV	64% (7 of 11)	85% (22 of 26)	0.74 (95% CI 0.57–0.87)
Combination of skewness and ECV	64% (7 of 11)	96% (25 of 26)	0.83 (95% CI 0.67–0.93)

## Discussion

Our study demonstrated that skewness from 3D iodine density histogram and ECV on dual-energy CT were significant factors for predicting PD-L1 expression. The combination of skewness and ECV showed good performance with AUC, sensitivity, and specificity of 0.83, 64%, and 96%, respectively. There is a trade-off between sensitivity and specificity, and it is ideal to optimally balance both. However, tests with high specificity can accurately identify patients with true diseases, helping provide appropriate treatment and care. The high specificity in the model of this study may be particularly valuable in non-invasively assessing the applicability of immune checkpoint inhibitors, especially in cases where surgery or biopsy is not feasible. Additionally, for biopsies that can only confirm a part of the lesion, CT imaging allows for the evaluation of the entire lesion, potentially leading to a more accurate diagnosis.

In this study, a significant relationship was found between the skewness of the iodine map in lung cancer and the expression of PD-L1. Generally, the skewness extracted in this study expresses the degree of distortion of the shape of the distribution, whether it is symmetrical or not, and is considered to be normally distributed when the skewness is 0. When the skewness is greater than 0, the distribution is skewed to the left, and when the skewness is less than 0, the distribution is skewed to the right. The magnitude of the absolute value of the skewness indicates the degree of asymmetry. Skewness ≤ − 2.3 in this study means that the left tail of the distribution is long. Although we can guess the possibility of PD-L1 expression to some extent from the distribution of the histogram of iodine maps, it is difficult to intuitively consider its reason. Several studies have investigated gene expression in tumors using texture analysis of CT values. Agazzi et al.^11^ reported that they performed CT texture analysis of 2-dimensional (2D) images of lung cancer and found an association between epidermal growth factor receptor (EGFR) mutations and CT histograms with left-sided tails (i.e., a negative value of skewness). In addition, the meta-analysis investigating the correlation of PD-L1 expression with EGFR mutations in lung cancer showed that high PD-L1 expression was associated with EGFR mutations^12^. These results suggest that the lower the skewness value obtained from CT texture analysis, the higher the PD-L1 expression may be. Overall, this is consistent with the results of the present study using texture analysis of iodine density, given the high correlation reported between CT values and iodine density^13^. Three-dimensional quantitative analysis offers more precise and reproducible results compared to 2D analysis, enabling better prediction of clinical outcomes^14^. Unlike 2D analysis, 3D analysis can also capture the entire tumor image, resulting in less observer bias. Therefore, the present study using 3D analysis has the potential to provide more accurate results than traditional 2D methods.

Regarding an association between ECV and PD-L1 expression in lung cancer, the present study showed that ECV was a significant indicator for predicting PD-L1. ECV measurement plays a central role in the assessment of tissue fibrosis and provides valuable insight into pathological processes associated with excessive extracellular matrix deposition. Fibrosis, characterized by abnormal accumulation of collagen and other extracellular matrix components, is a common feature of several organ diseases^7,8^. ECV is a promising tool for assessing the extent of fibrosis in tissues. A variety of cells, including immune cells, microvessels, and fibroblasts, exist in tumor tissue and form the microenvironment. This microenvironment is known to interact with tumor cells and play an important role in activities such as tumor growth and metastasis. In particular, fibroblasts within the tissue microenvironment, called CAFs, are known to play the most important role^15,16^. The induction of PD-L1 in carcinoma cells has been reported as a response to exposure to inflammatory cytokines, such as IFN-γ, and the activation of oncogenic pathways. Concurrently, CAFs release inflammatory cytokines and growth factors, subsequently initiating oncogenic pathways in carcinoma cells^15,16^. Inoue et al.^10^ demonstrated that CAFs indirectly influenced tumor immunity through increasing PD-L1 expression in lung adenocarcinoma cells. In other words, ECV may correlate with CAF within the tumor stroma and may be a predictive indicator of PD-L1 expression, which is in agreement with the present study.

PD-L1 expression is useful in predicting therapeutic response to ICI. High PD-L1 expression may indicate sensitivity to treatment with these agents. In general, PD-L1-positive patients may respond better to treatment with ICI than PD-L1-negative patients. PD-L1 expression levels can therefore guide the choice of treatment strategy and predict patient prognosis. In this study, skewness and ECV showed good diagnostic performance in predicting PD-L1 expression, with AUC values of 0.76 and 0.74 respectively, and even better with an AUC value of 0.83 when these two values were combined. Therefore, these two values might have the potential to be imaging biomarkers for the non-invasive prediction of therapeutic response to ICI. Considering that 3D CT analysis can capture the entire lesion unlike tissue biopsy, it is expected that the combination of tissue diagnosis and image diagnosis may lead to more accurate prediction of gene expression. Large-scale cohort studies will be essential for further validation and implementation in clinical practice.

There are several limitations to the study. First, this study included only a small number of cases. There might be overfitting and bias issues regarding multivariate analysis, especially as there are only 11 PD-L1 positive cases (TPS ≥ 50%). In addition, advanced lung cancers were not included. Second, because fibroblasts including CAF were not immunostained, the relationship between ECV and lung cancer fibrosis remains speculative. Although we found that ECV is useful for predicting PD-L1 expression in lung cancer, it would have been better to pathologically examine the relationship with CAF. Third, 3D iodine density mapping was evaluated using CT devices manufactured by a single company. While the outcomes of this study may have been affected by the CT device's performance, there have been few researches thus far analyzing iodine density obtained from three-dimensional DECT. We anticipate future clinical applications of this research. Fourth, in this study, a 3D analysis of lung nodules was conducted. However, in part-solid nodules, the iodine value in the ground-glass component is also computed. The iodine value in the ground-glass component may not accurately reflect a true value due to the impact of air and image noise. Therefore, further investigation may be necessary in the future. Sixth, conventional predictive models using texture analysis of original CT images, visual CT evaluation, or clinical findings were not evaluated in this study. Finally, equilibrium imaging is a technique that uses contrast agents commonly used in CT to assess ECV, but the timing of the delayed scan varies among previous reports^8,17,18^. In the present study, ECV was measured at 3 min after contrast material injection, which is the clinical protocol used in our hospital. There may be room to verify whether this is the optimal equilibrium phase for ECV measurement in lung cancer.

In conclusion, skewness and ECV from dual-energy CT in the 3 min late phase may predict high expression of PD-L1 in invasive pulmonary adenocarcinoma. Dual-energy CT may be a noninvasive method for chemotherapy strategy such as immune checkpoint inhibitors in invasive adenocarcinoma.

## Methods

### Patient

The present study was approved by the ethical review board of Osaka University Hospital (No. 23452) and the need for informed consent was waived due to the retrospective review of patient records and images. This study was conducted following the principles of the Declaration of Helsinki (as revised in 2013).

The study population consisted of 97 consecutive patients who had undergone dual-energy dynamic multiphase CT to examine a solitary lung nodule at one hospital from January 2019 to April 2020.

**Figure 3:**
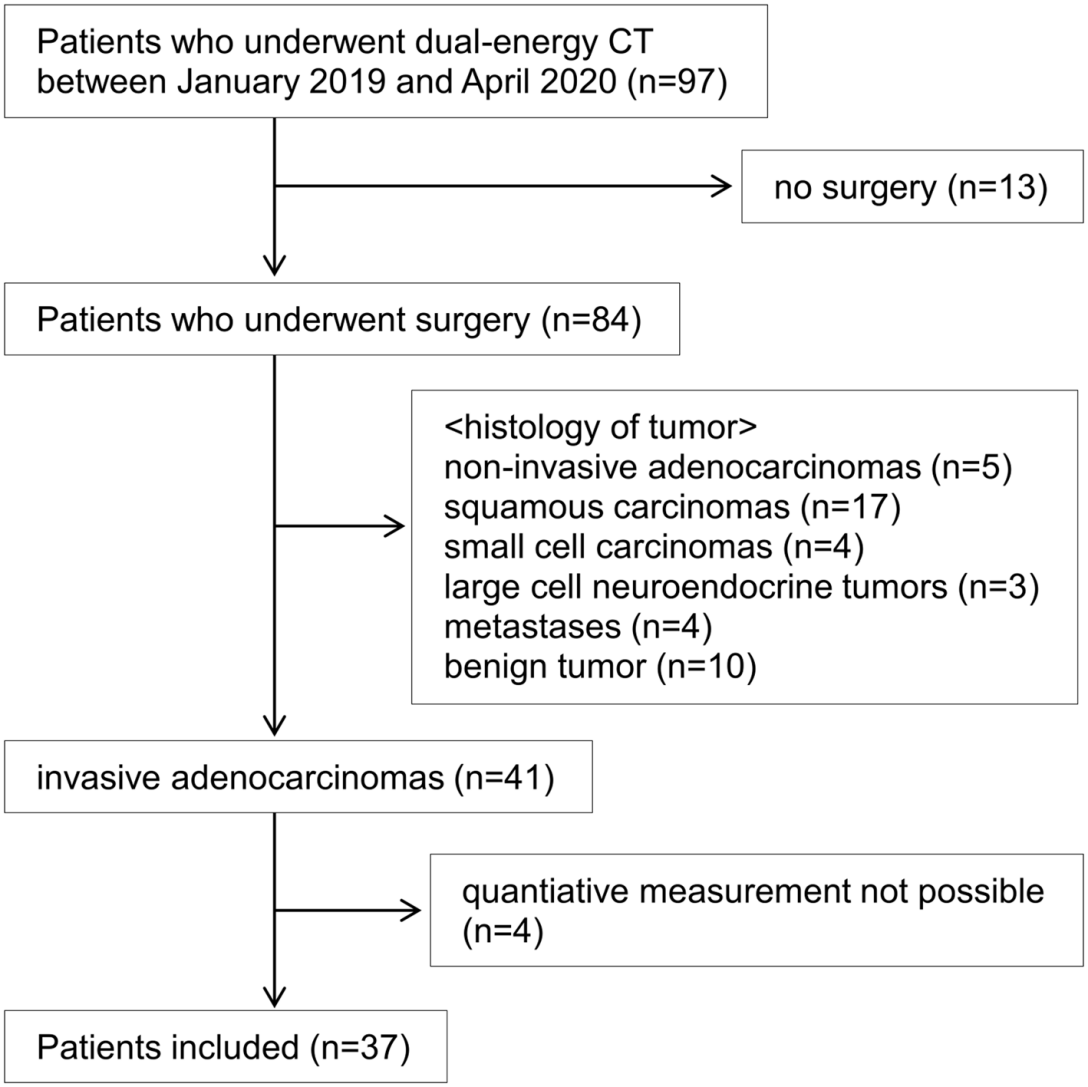
Flowchart of patient selection.

The inclusion criteria were as follows: (a) CT examination within 3 months before surgery; (b) operable patients with clinical stage I or II; (c) no previous treatment in the lungs or any other organ; (d) dual-energy dynamic multiphase CT was obtained; (e) age 20 years or older; (f) invasive adenocarcinoma. Of the 84 excluding patients with no surgery (n = 13), 43 were excluded because of non-invasive adenocarcinoma, and 4 because quantitative measurement was not possible (Fig. 3). Finally, 37 patients with invasive adenocarcinoma (age range, 38–85 years; median age, 71 years; and 22 men and 15 women) were entered into the present study.

### Dual-energy CT protocols

Dual-energy CT scans were performed on a Revolution CT (GE Healthcare, Milwaukee, WI, USA). CT protocol was as follows: matrix size, 512 × 512 pixels; X-ray voltage, fast kV switching between 80 and 140 kVp; field of view, 20 cm for targeted lung. Images were reconstructed into 1.25 mm sections using a standard kernel and 30% adaptive statistical iterative reconstruction. CT images targeted to the lung nodule were obtained before (unenhanced scan) and 3 min after contrast material (IOHEXOL with an iodine content of 300 mg/cc; Daiichi Sankyo company, limited, Tokyo, Japan) injection (3-min delayed scan). Contrast volume was determined by weight (2 ml/kg). The mean contrast volume and injection rate were 114.9 ± 17.5 ml (76—147 ml) and 1.93 ± 0.29 ml/s (1.3–2.5 ml/s), respectively. The radiation dose was as follows: CT dose index volume (CTDIvol), 37.4 ± 8.4 mGy; and dose-length product (DLP), 1426.4 ± 319.3 mGy cm.

### Image analysis

**Figure 4:**
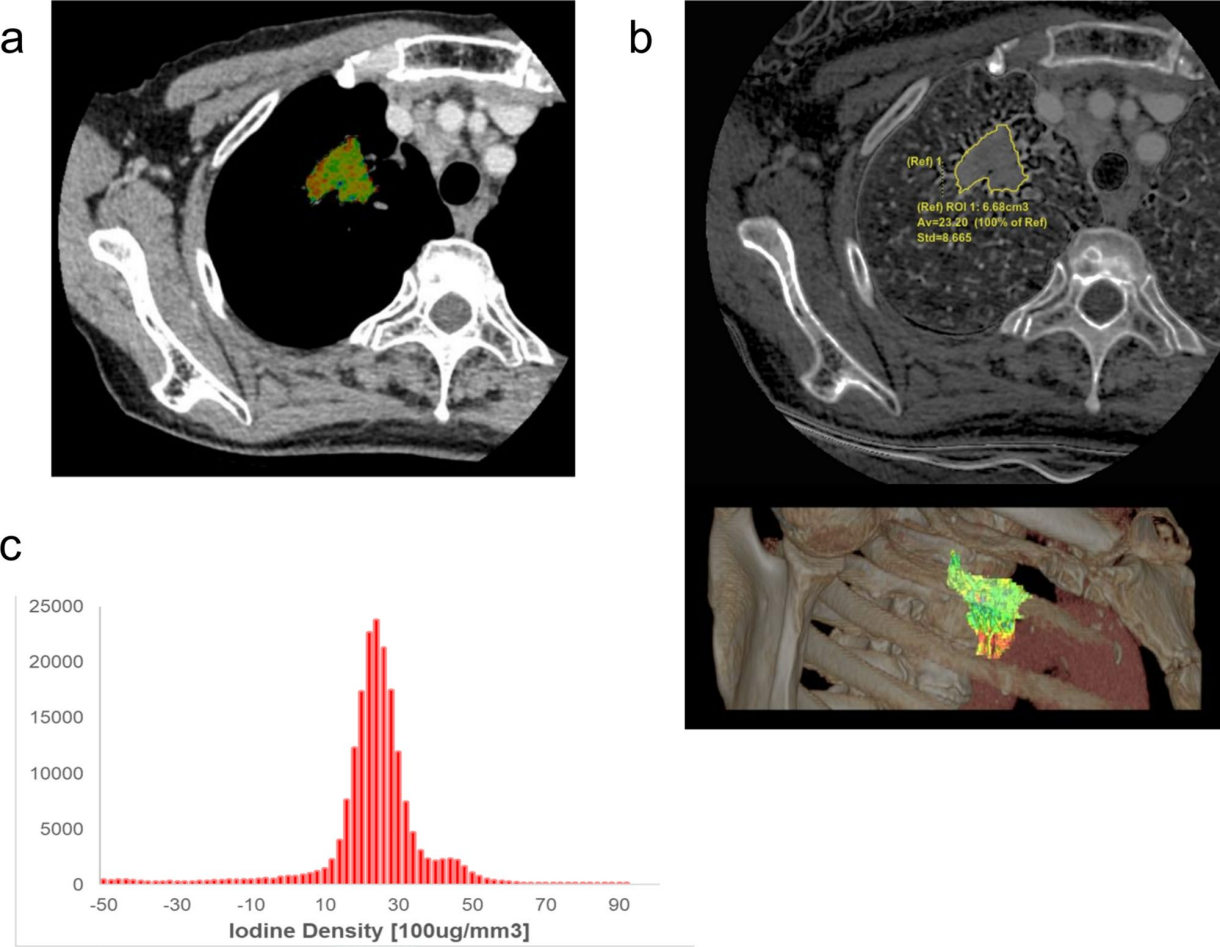
Texture features extracted from iodine map. Volumetry of each nodule was semi-automatically performed by including tumor margin as much as possible (**A**). 3D volume of interest (VOI) on tumor was extracted and it was applied to iodine density image (**B**). Texture features were extracted for each 3D iodine density histogram (**C**).

Image data (n = 37) were transferred to AW Volumeshare 7 (GE Healthcare). Tumor boundary was semi-automatically traced slice by slice using GSI Volume Viewer, and any inappropriate segmentation was corrected manually. The 3D volume of interest (VOI) on the tumor was extracted and it was applied to the iodine density image. VOI on 3D iodine density mapping was decided with the consensus of two chest radiologists in each nodule as large as possible to reduce bias due to tumor heterogeneity. For part-solid nodules, the measurements were obtained for the entire lesion, including ground-glass opacity. 7 texture features (max, min, median, mean, standard deviation, skewness, kurtosis) were extracted for each 3D iodine density histogram (Fig. 4).

Extracellular volume fraction (ECV) was also calculated. Assuming that the extracellular fluid iodine contrast agent is evenly distributed in the extracellular fluid space in the equilibrium phase, the ECV is obtained by correcting the ratio obtained from the tissue iodine value and the aortic iodine value, which are differenced in the equilibrium and non-enhanced phases on CT, by the hematocrit value. ECV was calculated as follows:

$$ {\text{ECV }}\left( \% \right) \, = \, \left( {{1 } - {\text{ hematocrit}}} \right) \, \times \, \left( {{\text{iodine}}\,{\text{ density}}\,{\text{ in }}\,{\text{each }}\,{\text{nodule}}} \right)/\left( {{\text{iodine}}\,{\text{ density}}\,{\text{ in }}\,{\text{each}}\,{\text{ aorta}}} \right) \, \times { 1}00. $$

### Immunohistochemistry

The PD-L1 immunostaining was performed by pathology specialists in our institution according to the guidelines^19^. The tissues fixed in 4% buffered formalin were processed and embedded in paraffin. The tissues were sectioned with 3-μm thick and dried onto slides overnight at 60 °C. After deparaffinization in xylene and rehydration for decreasing ethanol solutions, the slides were heated in 0.01 M citrate buffer (pH 6) in a Pascal pressurized heating chamber (Dako Glostrup, Denmark). The sections were incubated with the primary antibody (PD-L1 IHC 22C3 pharmDx, Dako) for 20 min. To detect primary antibody, Dako EnVision FLEX + Polymer Reagents, including a mouse linker, horseradish peroxidase polymer, diaminobenzidine chromogen (DAB), and DAB enhancer were added. The EnVision FLEX + Wash Buffer is applied for washing between each reaction step. After primary antibody detection, the slides are counterstained with hematoxylin and coverslipped. A negative control was performed by substituting the primary antibody with the mouse IgG isotype. With regard to PD-L1 scoring, the same pathologist evaluated a tumor proportion score (TPS, %) using PD-L1 immunostaining: PD-L1 high expression (TPS ≥ 50%) and low or negative expression (TPS < 50%).

### Statistical analysis

All statistical analyses were performed using commercially available software (MedCalc Version 20.115, Frank Schoonjans, Mariakerke, Belgium).

The relationship between age, sex, and pathological T descriptor distribution and PD-L1 expression was analyzed by the Mann–Whitney U test or chi-square test.

For each of 8 parameters (max, min, median, mean, standard deviation, skewness, kurtosis, and ECV), the cutoff value that yielded the largest difference in numbers of patients with and without PD-L1 expression (TPS ≥ 50% and TPS < 50%) was determined using the receiver-operating characteristic (ROC) method. Optimal thresholds were determined for each variable separately using the Youden index (the highest sum of sensitivity and specificity). The group that met the cutoff value condition was graded as score = 1 and the other group was graded as score = 0. The difference between the two groups was analyzed by the Mann–Whitney U test.

Associations between the expression of PD-L1 and each binary group designated by the cutoff value for the 8 parameters were evaluated by univariate logistic regression analysis. Significant parameters identified by univariate analysis were included in multiple logistic regression (stepwise method; a *P* value of 0.05 or less was used for entry into the model and a P value greater than 0.1 was selected for removal). Diagnostic performance for PD-L1 expression was evaluated by sensitivity, specificity, and an area under the receiver operator characteristic curve (AUC). *P* values < 0.05 were considered significant.

## Data Availability

The datasets used and analysed during the current study available from the corresponding author on reasonable request.
